# The Role of Fatigue in the Relationship Between Sleep and Concentration Among Online College Students

**DOI:** 10.3390/ijerph22111728

**Published:** 2025-11-15

**Authors:** Fethi Ahmet Inan, Deniz Unal, Fatemeh Marzban, Edwin Teye Sosi, Gail Alleyne Bayne

**Affiliations:** Educational Instructional Technology, Texas Tech University, Lubbock, TX 79409, USA; dnal@ttu.edu (D.U.); fatemeh.marzban@ttu.edu (F.M.); esosi@ttu.edu (E.T.S.);

**Keywords:** college students, mental health, fatigue, sleep, health behaviors, concentration, well-being, academic performance, cognitive function

## Abstract

Sleep deprivation is a common issue among college students, critically impairing their well-being and academic performance. This study specifically investigated the impact of sleep duration on concentration among online college students, a population with unique living situations and often irregular sleep patterns. Furthermore, it explored how this relationship is mediated by subjective physical and mental fatigue, providing a clearer understanding of the underlying process. An online survey assessed these variables using the Demographics and Background Questionnaire for sleep duration, the Student Mental Fatigue Survey (SMFS) for mental fatigue, and two subscales from the Checklist Individual Strength (CIS) for subjective physical fatigue and concentration. Path analysis revealed that both subjective physical and mental fatigue significantly and negatively predicted concentration, and that sleep duration positively influenced concentration indirectly by reducing both types of fatigue. The findings highlight the essential function of sleep in improving concentration. The results provide valuable guidance for developing targeted interventions to improve sleep quality and manage fatigue, which can directly promote mental and physical health, and academic success of this growing, often overlooked, online college student population.

## 1. Introduction

### 1.1. Background

College students are a vulnerable population particularly susceptible to poor sleep habits such as reduced sleep duration and inconsistent sleep schedules. Sleep is a vital element of general health and well-being, and crucial for healthy brain function [1]. The recommendation for college students is to get 7–9 h of sleep per night [2]. However, previous studies document that college students frequently experience disturbed or inadequate sleep [3]. American College Health Association (2025) reported that over 75% of college students have been getting inadequate sleep, over 61% reported daytime sleepiness three or more days a week, and over 20% stated that sleep issues had a detrimental effect on their academic performance [4]. Similarly, another study reported that 60% of students are not getting enough sleep, 75% of college students indicated periodic sleep problems, and 15% reported overall bad sleep quality [3].

Sleep patterns of young adults and college students are frequently disrupted by lifestyle, work pressure, and staying up late [5]. Academic pressure, technological use, and biological changes are all common causes of poor sleep in young adults. Studies have consistently indicated a relationship between sleep habits and academic performance. Previous research shows that better academic performance is linked to sleep habits such as longer sleep duration, higher quality sleep, and more consistent sleep [6]. Existing research demonstrates that both the procedural and declarative memories of learners are impacted by the quality of sleep [7,8].

As online learning becomes more prevalent, offering convenience in balancing commitments such as jobs and family, it can either improve sleep routines due to increased flexibility or worsen them due to irregular schedules and late-night study habits. It is anticipated that online students, especially those with multiple commitments, may be more prone to sleep problems due to the time required for additional coursework [9]. Conversely, the flexible scheduling of asynchronous online courses can allow students to better align academic work with their natural sleep–wake cycles [10]. Despite the rising number of online students, this group remains underrepresented and frequently overlooked in research focused on sleep and concentration [11]. According to a recent report [12], online enrollment has been significantly influenced by the need for flexibility, with about one-third of respondents enrolling in online programs to accommodate existing commitments like work and family. Their distinctive learning environment, which demands greater self-directed study, may increase pressure to balance academic responsibilities with personal commitments. Research indicates that students enrolled in online courses often experience challenges such as elevated anxiety levels, difficulties in maintaining their well-being, and issues related to managing academic workload and time [13]. Such circumstances can potentially worsen problems related to sleep deprivation and its effects on cognitive performance.

This study contributes to the existing literature by utilizing path analysis to investigate the intricate, mediated relationships between sleep duration, physical and mental fatigue, and concentration in the online college student population. While the broader effects of sleep on cognitive function are well-documented [14], this research offers a more detailed perspective on fatigue as a critical mediator in online learning environments. Specifically, this research delves into the specific pathways through which sleep duration influences concentration, highlighting the distinct roles of both subjective physical and mental fatigue. By focusing on online college students, this study addresses a unique demographic that may experience different sleep patterns and fatigue levels due to the nature of their environment. The findings have important implications not only for the academic research community but also for university policy makers for designing intervention strategies to improve the overall well-being of students. By explaining how sleep deprivation affects cognitive performance (concentration), the study provides guidance for the development of targeted support systems to address sleep-related problems and fatigue management. Ultimately, these insights enable the promotion of effective learning experiences without compromising mental or physical health, supporting academic achievement within the increasingly prevalent context of online education.

### 1.2. Sleep Duration and Fatigue

Sufficient sleep is crucial for human physical and mental well-being [15]. However, many college students report inadequate sleep, characterized by late bedtimes, insufficient duration, or inconsistent patterns [3,5]. Sleep deprivation is a primary cause of fatigue, encompassing both physical and mental aspects. Fatigue is the subjective feeling encompassing perceived lack of physical or mental capacity, or energy [16,17] and primarily assessed through self-reports and descriptions of subjective feelings of exhaustion, lack of motivation, or difficulty completing tasks [18,19]. While mental fatigue, also known as cognitive fatigue, refers to the subjective feeling of being mentally exhausted, physical fatigue is the subjective perception of being worn out and lacking energy [20]. Subjective physical fatigue is predominantly characterized by an individual’s self-reported, overall feeling of tiredness and exhaustion, encompassing a diminished sense of physical energy and the subjective perception of reduced capacity to sustain effort [21]. In contrast, mental fatigue is characterized by cognitive impairment, presenting as challenges with concentration, maintaining attention, and effective information processing due to diminished cognitive resources [22]. Extensive research, including empirical studies and sleep monitoring technologies, consistently shows that reduced sleep duration leads to pronounced levels of both types of fatigue. Therefore, we hypothesize the following relationships:

**H1.**  *Sleep duration directly negatively impacts subjective physical fatigue.*

Lack of sleep is a major contributing factor to subjective physical fatigue [23,24]. When individuals are sleep-deprived, their bodies lack the necessary rest and repair, leading to feelings of physical exhaustion and a perceived lack of energy [17,23]. Sleep monitoring technologies have been used to track this relationship, predicting that pronounced levels of sleep deprivation induce fatigue [25,26,27,28].

**H2.**  *Sleep duration directly negatively impacts mental fatigue.*

Similarly, sleep deprivation directly contributes to mental fatigue. Mental fatigue is characterized by cognitive impairment and is highly predicted by disturbed sleep [29]. Lack of sleep prevents neurons in the brain from resting and repairing, hindering their normal function, and leading to symptoms of cognitive issues [30,31]. Research provides substantial evidence of sleeplessness-induced mental fatigue among learners [32,33,34].

### 1.3. Fatigue and Concentration

Both mental and physical aspects of fatigue significantly impact a person’s ability to concentrate. This lack of concentration can hinder how well someone learns and acquires new knowledge [1,35,36,37] and can cause the brain to process information less efficiently thus leading to cognitive errors and biases [26,38,39]. For example, Shahriar and Koly found that many students had trouble concentrating in online classes, with fatigue being a key factor [40]. Mosleh et al. noted that poor concentration and memory issues are common for students experiencing fatigue [41]. In a qualitative study, Godara et al. reported that students often felt “zoned out” and had trouble sustaining attention during virtual meetings because of fatigue. Based on this evidence, the following hypotheses were proposed [42].

**H3.**  *Mental fatigue directly negatively impacts concentration.*

Mental fatigue, often stemming from sleepiness, has been found to impair cognitive functions [28,30,43]. It directly negatively impacts concentration, which is crucial for enhancing cognitive functioning and acquiring knowledge [1,36]. When mentally fatigued, individuals find it harder to focus and maintain attention, leading to decreased performance in challenging activities [35,37]. For instance, Mosleh et al. identified cognitive impairments as part of the fatigue experience, with 18% of students reporting poor concentration and 23.3% experiencing difficulty recalling recent information, highlighting these symptoms as a source of mental fatigue in learning contexts [41].

**H4.**  *Subjective physical fatigue directly negatively impacts concentration.*

Subjective physical fatigue also directly and negatively impacts concentration. This type of fatigue is characterized by the muscles’ failure to sustain the necessary strength [19] and is more outwardly apparent than mental fatigue [44]. Similarly to mental fatigue, subjective physical fatigue causes physiological difficulties that impair concentration, slows reaction times, and can disturb attention [45]. High levels of both mental and subjective physical fatigue can cause the brain to shift to a more energy-efficient mode of information processing, leading to cognitive biases and errors, and compromising neuro-cognitive functions [26,38,39].

### 1.4. Sleep and Concentration

The impact of sleep on cognitive function has been established. While studies indicated poor sleep’s impact on student cognitive function and academic outcomes, the mechanism to explain the relationship and how sleep leads to cognitive impairments such as reduced concentration involves the mediating role of both physical and mental fatigue. Sleep deprivation affects the brain’s function by reducing its energy resources. Extended periods of wakefulness can lower levels of glycogen and other energy sources, resulting in less energy available for optimal brain functioning [46]. This reduction in energy can lead to both physical and mental fatigue [47,48]. This fatigue, in turn, causes performance deficiencies including impaired concentration [49]. Specifically, sleep loss compromises the functioning of the prefrontal cortex, which is highly sensitive to sleep and crucial for cognition and memory [50]. This compromised brain function due to fatigue then hinders the ability to focus, analyze, and make decisions, thus directly impacting concentration. Therefore, the following hypothesis was proposed.

**H5.**  *Sleep duration indirectly positively impacts concentration through subjective physical fatigue and mental fatigue.*

Sleep is essential for the brain’s ability to focus, analyze, and respond to input. Sleep plays a role in supporting the brain’s capacity to focus, analyze, and process information. Research indicates that during sleep deprivation, the brain shows reduced metabolic activity, including lower production and utilization of adenosine triphosphate (ATP), which is the primary energy source for cells. This reduction provides an explanation for the fatigue experienced when adequate sleep is not obtained [50]. When sleep-deprived, the neural energy reserves needed for high-quality performance become insufficient, leading to energy resource loss that presents as subjective physical fatigue or mental fatigue, which in turn causes performance deficiencies like difficulty concentrating and making decisions [23,51]. This mediating effect highlights how adequate sleep, by mitigating fatigue, plays a crucial role in maintaining optimal concentration and cognitive performance.

## 2. Materials and Methods

### 2.1. Participants

Institutional review board approval was obtained from the Human Research Protection Program of Texas Tech University (504953, approved on 02 November 2016). All procedures performed in this study were in accordance with the ethical standards of the institutional and national research committee and with the 1964 Helsinki Declaration and its later amendments or comparable ethical standards. The participants were recruited for the study by Qualtrics Panels, LLC. All participants provided informed consent prior to participation. Participants were given an online survey and asked to complete a series of questions, including their self-assessment of their level of mental fatigue while participating in an online course.

The study included 618 undergraduate students with complete data. Participants, aged 18 or older and enrolled in a fully online course at U.S. 4-year universities, were recruited via the Qualtrics Sample Panel using a proportional and randomized selection approach. The majority were female (83.56%) aged 18–50 years (M = 26.85, SD = 7.43). Participants self-identified with the following ethnicities: White (65.98%), Black/African American (15.07%), Hispanic/Latino (10.73%), Asian (3.43%), and Other/Undisclosed (2.51%). Most students are in their first year (26.43%) or second year (40.48%) of college.

### 2.2. Instruments

Demographics and Background Questionnaire: This questionnaire gathered information regarding participants’ demographic details and background characteristics. In particular, it included a single-item question asking students to self-report the average number of hours of sleep they receive each night to assess the sleep duration. Considering the scope of the study and constraints on survey length, the use of a single-item measure provided a pragmatic means of assessment within a non-clinical, educational context [52,53]. This approach aligns with previous large-scale data collections, such as the National Health and Nutrition Examination Survey, which also employed a single-item measure of sleep duration [53]. Additionally, previous research indicates that single-item questions for measuring sleep quality can yield valid and reliable outcomes within clinical populations [54,55,56].

Student Mental Fatigue Survey (SMFS): SMFS was used to measure participants’ self-reported perceptions of mental fatigue experienced while engaging in online coursework. This validated instrument was originally developed across three distinct stages: identification of constructs and development of subscale items, expert review and validation, and scale analysis and optimization [22]. The instrument consists of 8 items; each rated on a 5-point Likert scale (from 1 = strongly disagree to 5 = strongly agree). An average score was computed for the survey, with higher scores corresponding to higher levels of mental fatigue. The SMFS has been utilized in various studies [57,58] and demonstrated high internal consistency in a prior validation study (Cronbach’s α = 0.91).

Checklist Individual Strength (CIS): The instrument is a self-report questionnaire originally developed and validated to measure four dimensions of fatigue [59]. The instrument is widely used in research studies, including those relevant to educational contexts [60,61]. For this study, two subscales from the CIS were used: the Subjective Fatigue Subscale and the Concentration Subscale. Participants rated each item on a 7-point Likert scale (1 = “Yes, that is true” to 7 = “No, that is not true”).

- Subjective Fatigue Subscale (8 items): This subscale measures participants’ self-reported experience of general tiredness, exhaustion, and physical energy levels. A higher average scale score, determined by taking the mean of eight items (some reverse-scored), indicates an increased level of perceived subjective fatigue. The subscale has demonstrated high internal consistency in the validation study (Cronbach’s α = 0.88) [59].
- Concentration Subscale (5 items): This subscale assesses participants’ self-reported ability to focus and maintain attention. A higher average score across the five items (some reverse-scored) indicates stronger concentration. The original validation study also reported high reliability for this subscale (Cronbach’s α = 0.92) [59].

### 2.3. Data Analysis

Path analysis was used to examine the hypothesized relationships between sleep, mental fatigue, subjective physical fatigue, and concentration. The model specified sleep (hours of sleep) as an exogenous variable that directly impacted both subjective fatigue and mental fatigue. Both subjective fatigue and mental fatigue were specified as mediators of the impact of sleep on concentration and also as directly impacting concentration. The covariance between subjective physical fatigue and mental fatigue was modeled. All analyses were conducted using Mplus 8.11 (Muthen & Muthen, Los Angeles, USA) and JASP statistical software (Version 0.95.3, JASP Team, Amsterdam, The Netherlands). The model was estimated using the Maximum Likelihood (ML) estimator, and standard errors were calculated using the bootstrapping method with 1000 iterations.

## 3. Results

### 3.1. Descriptive Statistics [f1]

An examination of the descriptive analysis results indicated that, on average, participants reported approximately 6 h of sleep (M = 5.97, SD = 1.25). In terms of perceived mental fatigue (M = 3.19, SD = 0.93), scores suggested participants experienced a moderate level of mental fatigue. For subjective physical fatigue, the mean score was 4.43 (SD = 1.31), indicating a moderate level of perceived physical fatigue. Concentration levels averaged 3.94 (SD = 1.30), suggesting a moderate self-reported concentration ability. Table 1 below presents a descriptive (means and standard deviations) overview of the variables involved in the path model. Correlations between each variable are shown at the intersection of their respective rows and columns.

### 3.2. Path Model Estimates 

In the path analysis, the factors examined included sleep duration as an exogenous variable, and subjective physical fatigue, mental fatigue, and concentration as endogenous variables. Consistent with the proposed fatigue mediation model, subjective physical fatigue and mental fatigue served as mediator variables. The covariance between subjective physical fatigue and mental fatigue was explicitly modeled within the path analysis. The size and direction of effects were determined from standardized path coefficients (betas) obtained using Mplus Version 8.11 [62]. Indirect effects were estimated, and their statistical significance was assessed by calculating standard errors and *p*-values using bootstrap confidence intervals (1000 replications). Statistical significance for all direct and indirect effects was evaluated using *p*-values, with effects considered significant at an alpha level of 0.01. In this context, a “positive impact” signifies that an increase in one variable corresponds to an increase in others, while a “negative impact” indicates that one variable increases as another decreases. These terms describe the direction of interaction, not the overall desirability of the outcome. The specified path model demonstrated excellent fit to the data: CFI = 1.00; TLI = 1.00; RMSEA = 0.000; and SRMR = 0.009. The Chi-Square test of model fit was not statistically significant (χ^2^(1) =0.981, *p* = 0.3219), further indicating good model fit. Furthermore, the AIC and BIC values were 3698.561 and 3743.465, respectively.

The hypothesized factors in the model explained approximately 46.1% of the variance in students’ concentration ($R^{2}$= 0.461, *p* < 0.001). Consistent with the model, both subjective physical fatigue and mental fatigue showed significant negative direct effects on concentration. Specifically, subjective physical fatigue had a strong negative direct effect (standardized β = −0.505, *p* < 0.001), and mental fatigue had a significant negative direct effect (standardized β = −0.268, *p* < 0.001) on concentration.

The model significantly predicted the mediator variables, explaining 9.5% of the variance for subjective physical fatigue ($R^{2}$ = 0.095, *p =* 0.001) and 8.2% of variance for mental fatigue ($R^{2}$ = 0.082, *p* = 0.001). Sleep duration had a significant negative direct effect on mental fatigue (standardized β = −0.287, *p* < 0.001) and a significant negative direct effect on subjective physical fatigue (standardized β = −0.308, *p* < 0.001).

**Figure 1:**
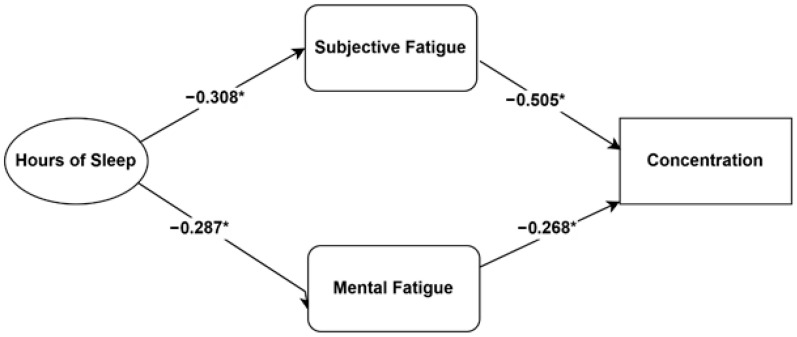
Path Model Estimates. Note: * *p* < 0.01.

The path analysis revealed significant indirect (mediation) effects. Sleep duration had a significant positive indirect effect on concentration (standardized β = 0.232, *p* < 0.001). This indirect effect was channeled through both mediators: The indirect effect of sleep duration on concentration via subjective physical fatigue was significant (standardized β = 0.155, *p* < 0.001). The indirect effect of sleep duration on concentration via mental fatigue was also significant (standardized β = 0.077, *p* < 0.001). Further details regarding the standardized regression coefficients (β) and the coefficients of determination ($R^{2}$) are presented in Table 2, with the estimated relationships among variables visually depicted in Figure 1.

## 4. Discussion

This study investigated the intricate relationships among sleep duration, subjective physical fatigue, mental fatigue, and concentration in online college students. Building on previous research, a model was proposed to explain the mechanism of how sleep impacts cognitive function. Our findings provide compelling evidence for the proposed model, highlighting the significant role of fatigue as a mediator between sleep and concentration.

Consistent with our hypotheses, shorter sleep durations were directly and negatively associated with both subjective physical fatigue and mental fatigue. This aligns with extensive previous research recognizing sleep deprivation as a major contributor to both types of fatigue [23,24]. This is further supported by the restorative theory of sleep, which explains that the body and brain rely on sleep to recover from the physiological and cognitive demands of daily life [63,64]. During sleep, essential restorative processes occur, such as the repair of tissues, replenishment of energy stores, and consolidation of memories [65,66]. Insufficient sleep disrupts these restorative processes, leading to an accumulation of metabolic byproducts and a depletion of neurotransmitters necessary for optimal functioning, thus manifesting as both physical and mental fatigue [15,35,50]. Therefore, when online college students experience insufficient sleep, their bodies and minds are deprived of essential rest and repair, leading to feelings of physical and mental exhaustion and impaired cognitive function [67]. Lack of sleep not only impairs essential cognitive processes, but it can also manifest as daytime sleepiness and impaired memory consolidation, which can weaken a learner’s ability to grasp new material. In the long term, reduced cognitive abilities could lead to lower academic expectations and performance [68]. Among online student populations, insufficient sleep is prevalent and often arises from the need for self-regulation in learning while simultaneously managing family and work responsibilities [69]. This pattern typically results in decreased mental and physical health. To cope, online students may rely on energy drinks to alleviate fatigue [70], develop irregular sleep schedules [71], and decrease attentiveness to academic materials and activities, which can necessitate extended time to complete tasks [72].

Our findings strongly support the direct negative impact of both mental fatigue and subjective physical fatigue on concentration. This is consistent with prior research indicating that increased fatigue leads to a decline in cognitive functions, including concentration [35,73]. For learning tasks, increased mental fatigue can disturb cognitive functions because the effort to focus on relevant stimuli and suppress irrelevant stimuli depletes resources, leading to fatigue that weakens self-regulation mechanisms and attention [35]. Students experiencing mental fatigue reported difficulties in focusing and maintaining attention, affecting their ability to complete tasks [36,73,74]. Similarly, subjective physical fatigue, characterized by muscle weakness and a general feeling of being worn out, also impaired concentration, slowed reaction times, and dispersed attention [45]. Interestingly, subjective physical fatigue exhibited a stronger negative direct impact on concentration compared to mental fatigue. This might be attributed to the more apparent nature of physical tiredness compared to the cumulative and less obvious depletion of cognitive resources associated with mental fatigue [44]. These combined effects may be even more pronounced among online college students, as their extensive screen exposure, limited physical activity, and the demands of self-regulated learning can exacerbate both mental and physical fatigue [75,76,77].

One of the key findings of this study is the significant indirect positive impact of sleep duration on concentration, mediated through subjective physical fatigue and mental fatigue. This indicates that adequate sleep improves concentration by reducing both types of fatigue. The model accounted for approximately 46.1% of the variance in concentration, indicating a moderate effect and suggesting that additional factors beyond sleep and fatigue may also influence online student concentration. Sleep is a biological necessity for optimal brain function, including the ability to focus, analyze, and respond to input [78]. Sleep deprivation impacts the body’s regulation of energy storage and control. When an individual is sleep-deprived, the brain’s energy supply is reduced, resulting in decreased energy resources that can present as physical and mental fatigue [23,79]. During wakefulness, elevated brain activity necessitates increased energy production, leading to the processing of stored energy sources (e.g., glycogen), which in turn generates oxidants that accumulate in the brain [47]. Sleep is crucial for the removal of these accumulated oxidants and for restoring glycogen reserves, which serve as the principal energy source for the brain [23]. When sleep is restricted, glycogen levels remain low, leading to fatigue as the brain lacks sufficient energy for high-quality cognitive performance [46]. This fatigue, in turn, causes performance deficiencies, including difficulty concentrating and making decisions [23,51]. Our results emphasize that by mitigating fatigue, sufficient sleep plays a crucial role in maintaining optimal concentration and cognitive performance in online college students.

### Limitations

While this study offers valuable insights into the relationships between sleep duration, fatigue, and concentration in online learning, it is subject to several limitations that should be acknowledged. This study’s primary limitation is its reliance on a cross-sectional research design. Data collected at a single point in time inherently limits our ability to establish definitive causal relationships between sleep, fatigue, and concentration. While path analysis was employed to examine the hypothesized model, this method only indicates associations and potential mechanisms, not a finalized proof of causation [80]. Future research would greatly benefit from employing longitudinal designs to investigate how fatigue and concentration fluctuate throughout a semester and to track the long-term effects of sleep patterns on academic outcomes.

Additionally, the reliance on self-reported measures introduces potential bias. Specifically, in this study, sleep duration was assessed using a self-reported single item. Self-reported sleep duration is a common measure, and one-item question is a widely employed preliminary measure in large-scale survey research [53,81,82]. Studies show a strong correlation between subjective estimates and objective sleep duration, though subjective reports often overestimate objective sleep duration [82,83]. Future studies could enhance the robustness of sleep assessment and capture the multidimensional aspects of sleep by incorporating more comprehensive subjective scales, such as Pittsburg Sleep Quality Index [54], sleep diaries where participants log their sleep for a certain number of days [54], or utilizing objective measures such as wearable sleep-tracking devices [84]. However, collecting such objective, detailed sleep data presents logistical challenges and potential privacy concerns that are not always feasible in broad panel-based studies [85].

The study’s findings have limited generalizability due to the sample characteristics. The participants were primarily from the United States, and the sample exhibited a disproportionately high number of female participants. This gender imbalance is likely a reflection of two factors: the generally higher enrollment of women in online higher education institutions and a greater tendency for female students to participate in surveys [86,87]. This high representation may limit the direct applicability of the findings to more diverse populations. Future studies should proactively employ stratified sampling methods to ensure a more equitable distribution of student demographic characteristics, thereby enhancing the generalizability of the results to a broader online college student.

Another limitation of this study lies in the uncontrolled influence of various individual and lifestyle factors that could confound the observed relationships. Because this was a large-scale panel survey, it was beyond our scope to thoroughly examine several variables that may affect sleep duration, fatigue, and concentration. The findings indicate a limited contribution of sleep duration in explaining the fatigue outcome, with relatively low R^2^ values for mental fatigue (8.2%) and physical fatigue (9.5%). This suggests that additional unmeasured factors, such as stress, workload intensity, individual coping strategies, environmental conditions, and lifestyle habits, may also significantly influence fatigue levels [88,89,90]. Additionally, students’ overall health status, including pre-existing medical conditions and medication history, which can impact physiological and cognitive function, were not measured. Furthermore, individual characteristics such as academic abilities, self-regulation skills, coping mechanisms, and other concurrent commitments were not assessed, yet these are critical in determining how students manage their time and workload in online learning. The omission of these diverse confounding factors may limit the ability to isolate the exact effect of sleep duration, indicating a crucial direction for future research to control these variables to gain a more precise understanding of the dynamic interplay between sleep, fatigue, and concentration.

## 5. Conclusions

This study aimed to explore the intricate relationships between sleep duration, subjective physical fatigue, mental fatigue, and concentration among online undergraduate students. Our proposed path model, a novel framework in this context, successfully illuminated how these constructs interact. The findings consistently supported the model, demonstrating that sleep plays a crucial role in cognitive function, specifically concentration, primarily through its influence on fatigue. Specifically, shorter sleep durations were found to lead to increased subjective physical and mental fatigue, which in turn negatively impacted student concentration. The concerning prevalence of insufficient sleep among undergraduate students, often below the recommended seven hours per night [3,69,91], highlights the significant implications of these findings for their academic and overall well-being.

The study findings underscore the critical importance of promoting healthy sleep habits to mitigate fatigue and enhance concentration among online college students. Institutions and educators must implement comprehensive, targeted strategies to better support online students. One important intervention is to develop a mentally friendly online learning environment [92]. Given that excessive course workload is a significant contributor to both sleep deprivation and fatigue, it is essential for institutions to design courses that do not exacerbate these issues. This can be achieved by integrating strategies that reduce cognitive load and enhance engagement. Specific design tactics include creating user-friendly learning platforms where navigation is intuitive, ensuring the course content is manageable, and actively engaging students with instructional activities [93]. Furthermore, utilizing engaging instructional activities, such as project-based learning, that can promote student motivation and engagement [94]. Considering the motivation aspects of student learning, educators should prioritize aligning course content and activities with students’ personal and professional objectives to enhance perceived course value and foster meaningful engagement [95].

Another crucial aspect involves strengthening the availability of mental health and well-being support. Institutions should provide tailored mental health support services specifically designed for online students, addressing issues related to stress, anxiety, and isolation that are unique to the virtual learning environment. Since online learning can sometimes lead to feelings of isolation compared to traditional settings, offering opportunities for social interaction is also important, as robust social support systems influence well-being and stress levels [96]. Additionally, providing accessible resources like counseling services for students experiencing persistent sleep problems and offering workshops on stress management techniques (e.g., mindfulness, relaxation exercises) can empower students to cope with academic and personal pressures that frequently disrupt sleep [97].

Finally, promoting healthy sleep habits through dedicated interventions and strengthening academic support are vital. This involves implementing strategies like sleep counseling programs to educate students on sleep hygiene and offering readily available academic support resources to help students manage their coursework effectively, thereby reducing academic stress that can contribute to sleep problems [98]. Furthermore, institutions should leverage technology to support student self-monitoring. This could include recommending or providing access to online health platforms, sleep tracking apps, or educational modules focused on sleep hygiene and fatigue recognition [99]. By making use of these readily available digital tools, students can gain greater awareness of their sleep patterns and fatigue levels, empowering them to take preventative action and seek support when necessary.

## Figures and Tables

**Table 1:** Correlations, Means, and Standard Deviations (*N* = 438).

Variables	M	SD	1. Hours of Sleep	2. Subjective Physical Fatigue	3. Mental Fatigue	4. Concentration
1. Hours of Sleep	5.97	1.25	1			
2. Subjective Physical Fatigue	4.43	1.31	−0.31	1		
3. Mental Fatigue	3.19	0.93	−0.29	0.50	1	
4. Concentration	3.94	1.30	0.23	−0.64	−0.52	1

**Table 2:** Direct and Indirect Effects of the Factors (Standardized Coefficients).

	Endogenous (Dependent) Variables								
Variables	Subjective Physical Fatigue			Mental Fatigue			Concentration		
	Direct	Indirect	Total	Direct	Indirect	Total	Direct	Indirect	Total
Sleep Duration	−0.308 *			−0.287 *				0.232 *	0.232 *
Subjective Physical Fatigue							−0.505 *		
Mental Fatigue							−0.268 *		
$R^{2}$	0.095 *			0.082 *			0.461 *		

Note: * *p* < 0.01.

## Data Availability

The data that support the findings of this study are available from the corresponding author upon reasonable request.
